# Innate lymphoid cells exhibited IL-17-expressing phenotype in active tuberculosis disease

**DOI:** 10.1186/s12890-021-01678-1

**Published:** 2021-10-12

**Authors:** Linyue Pan, Xiaoli Chen, Xuanqi Liu, Wenjia Qiu, Yunhuan Liu, Weiping Jiang, Yang Zheng, Yan Mou, Wei Xu, Xiangyang Li, Haiyan Ge, Huili Zhu

**Affiliations:** 1 Department of Respiratory and Critical Care Medicine, The Affiliated Huadong Hospital of Fudan University, 221 West Yan’an Road, Shanghai, 200040 China; 2 Department of Respiratory and Critical Care Medicine, Zhongshan Hospital, Fudan University, Shanghai, China; 3 Department of Tuberculosis, the Sixth People’s Hospital of Nantong, Jiangsu, China; 4 Department of Geriatrics, The First Affiliated Hospital of Nanjing Medical University, Nanjing, China

**Keywords:** Tuberculosis, ILCs, IL-17, IL-23, DCs

## Abstract

**Background:**

Innate lymphoid cells (ILCs), as an important group of innate immunity, could respond rapidly to *Mycobacterium tuberculosis* (Mtb) infection. In this research, we studied the phenotypic changes of circulatory ILCs in active tuberculosis (TB) disease.

**Methods:**

We recruited 40 patients with active Mtb infection (TB group) and 41 healthy subjects (NC group), and collected their clinical information and peripheral blood. Circulating ILCs, ILC subsets, dendritic cells (DCs), macrophages, and the production of cytokines in ILCs were tested by flow cytometry (FCM). Enzyme-linked immunosorbent assay (ELISA) was used to detect plasma IL-23.

**Results:**

Compared with healthy control, total ILCs (0.73% vs. 0.42%, *P* = 0.0019), ILC1 (0.55% vs. 0.31%, *P* = 0.0024) and CD117^+^ ILC2 (0.02% vs. 0.01%, *P* = 0.0267) were upregulated in TB group. The total IL-17^+^ lymphocytes were elevated (3.83% vs. 1.76%, *P* = 0.0006) while the IL-22^+^ lymphocytes remained unchanged. Within ILC subsets, ILC3, CD117^+^ ILC2 and ILC1 in TB group all expressed increased IL-17 (15.15% vs. 4.55%, 19.01% vs. 4.57%, 8.79% vs. 3.87%, *P* < 0.0001) but similar IL-22 comparing with healthy control. TB group had more plasma IL-23 than NC group (7.551 vs. 5.564 pg/mL, *P* = 0.0557). Plasma IL-23 in TB group was positively correlated to IL-17^+^ ILC3 (*r* = 0.4435, *P* = 0.0141), IL-17^+^CD117^+^ ILC2 (*r* = 0.5385, *P* = 0.0021) and IL-17^+^ ILC1(*r* = 0.3719, *P* = 0.0430). TB group also had elevated DCs (9.35% vs. 6.49%, *P* < 0.0001) while macrophages remained unchanged. Within TB group, higher proportion of IL-17^+^ ILCs was related to severer inflammatory status and poorer clinical condition.

**Conclusions:**

In active TB disease, circulatory ILCs were upregulated and exhibited IL-17-expressing phenotype. This may expand the understanding of immune reaction to Mtb infection.

**Supplementary Information:**

The online version contains supplementary material available at 10.1186/s12890-021-01678-1.

## Introduction

Globally, there were an estimated 10.0 million tuberculosis (TB)-infected patients and an estimated 1.2 million TB deaths in 2019 [1]. Controlling tuberculosis which is infected by *Mycobacterium tuberculosis* (Mtb) remains a thorny issue.

Recently, innate immunity had gained much concerns in controlling a certain dose of Mtb without adaptive immunity engaged in mouse experiment [2]. As a microbicidal killer, bacille Calmette–Guérin (BCG)-trained macrophages were qualified to eradicate Mtb, with epigenetic modifications inherited from their progenitors [2]. New insight into the importance of innate immunity in Mtb infection is shared.

As another important group of innate immunity, innate lymphoid cells (ILCs) were also demonstrated to play a significant role in Mtb infection both in human and mouse [3, 4]. Generally, ILCs could be divided into two types, cytotoxic ILCs, namely natural killer cells (NK cells), and the other type helper ILCs. Helper ILCs consist of three major subgroups mirroring the classification of T helper cells. ILC1, the producer of interferon-γ (IFN-γ), is related to type 1 immune response (Th1); ILC2 is defined by cytokines profile of IL-5, IL-13, IL-4 and promoting type 2 immune (Th2); ILC3 is considered akin to T helper 17 cells (Th17) with expression of IL-17 and IL-22 [5]. However, this categorization was challenged by their unstable expression of cytokines and transcriptional factors, thereby introducing a new concept termed “plasticity” of ILCs [5]. For example, ILC2 was reported to produce IFN-γ [6] and IL-17, IL-22 [7, 8] under certain circumstances. A conversion from ILC1 to ILC3 was also detected in lung cancer [9]. This phenotypic flexibility probably was the strategy of host immunity evolved for various stimulator, keeping a timely and fast response. A study reported that ILC3 mediated early protection against Mtb [3] by migrating into primary infected area in lung via C-X-C chemokine receptor type 5 (CXCR5) and C-X-C motif ligand 13 (CXCL13), with boosted secretion of IL-17 and IL-22. Another mouse experiment found an intranasal BCG-induced accumulation of ILC1 and production of IFN-γ from ILC1 in lung and lymph nodes [4]. Still, how ILCs adjust their functional phenotype in response to Mtb had not been fully elucidated.

Th17 immunity, includes effector cytokines IL-17, IL-22, and stimulator cytokine IL-23. They all had been indicated to be protective factors in mouse Mtb model, regardless of their innate or adaptive origin [10–13]. However, others found that IL-23 was dispensable for Mtb infection and IL-17 even led to tissue damage by regulating matrix metalloproteinase activity [14, 15]. So far, the role of Th17-like immunity in Mtb infection, such as Th17-like ILCs were rarely known.

Data showed that only 5–10% latent infection of Mtb could progress into active infections, which signified a failed combat between host immunity and pathogen [16]. Our study focused on the active Mtb infection, and testified the numerical and phenotypic changes of ILCs (helper ILCs, same below) in response to Mtb. When compared with healthy control group, circulating ILCs had increased, among which CD117^+^ ILC2 and ILC1 had evident upregulation. In addition, ILC3, CD117^+^ ILC2 and ILC1 all had promoted ability to produce IL-17 in TB group. The ability to produce IL-17 of ILC3, CD117^+^ ILC2 and ILC1 all had a positive correlation to plasma IL-23 in active Mtb-infected patients. DCs were also upregulated in TB group, which could be the potential source of IL-23. Interestingly, we also found that higher proportion of IL-17^+^ ILCs was associated to severer inflammation and more clinical symptoms. These results may provide some clues for the exploration into the role of ILCs in active TB disease.

## Materials and methods

### Patients

Including 41 healthy subjects (aged 47.49 ± 10.56, female/male = 18/23), 40 active Mtb-infected patients (aged 49.13 ± 19.49, female/male = 10/30; diagnosed by sputum smears or culture method) without anti-Mtb treatment, were recruited from Huadong Hospital Affiliated to Fudan University and the Sixth People’s Hospital of Nantong. The study was approved by Clinical Research Ethics Committee of Huadong Hospital. Clinical information, laboratory test results, blood and written informed consents were collected from each patient.

### Flow cytometric analyses and gating strategy

In the first and second assays (Additional file 1: Fig. S1A), 200 μL whole blood was stimulated with phorbol-12-myristate 13-acetate (PMA, Sigma, MO, USA), ionomycin and Brefeldin A (Biolengend, CA, USA) at 37 °C for 4 h for cell activation. After that, it was incubated protected from light with BV510-viability kit (Zombie Aqua™ Fixable Viability Kit, Biolengend) at room temperature (RT) for 10 min first and then other surface antibody mixture for another 20 min: anti-CD45-APC/Fire 750 (clone HI30, 304061), anti-Lineage Cocktail-FITC (CD3, CD14, CD16, CD19, CD20, CD56, clone UCHT1, HCD14, 3G8, HIB19, 2H7, HCD56, 348801), anti-CD127-BV421 (clone A019D5, 351310), anti-CD294-PerCP/Cy5.5 (clone BM16, 350116), anti-CD117-PE/Cy7 (clone 104D2, 313211). After incubation, it was lysed by lysing buffer (Biolengend) for 15 min at RT and centrifuged 500×*g* for 5 min. The cell pallet was then fixed with fixation buffer (Biolengend) at RT for 30 min and washed by 1 mL intracellular staining permeabilization wash buffer (Biolengend). The cell suspension in 50 μL permeabilization wash buffer was stained with antibody to cytoplasmic antigen for 30 min again. The first assay intracellular antibody included anti-IL-17A-PE (clone BL168, 512306) and anti-IL-22-APC (clone 2G12A41, 366705); the second assay included anti-IFN-γ-PE (clone 4S.B3, 502508) and anti-IL-5-APC (clone TRFK5, 504305). After washing by 2 mL permeabilization wash buffer, the cell pellet was resuspended in 0.5 mL PBS and was for the further analyses. Helper ILCs were gated as CD45^+^Lineage^−^CD127^+^ cells; ILC1 was CD294^−^CD117^−^ ILCs; ILC3 was CD294^−^CD117^+^ ILCs, and the CD294^+^ ILCs cells were the total ILC2, which consisting of CD117^+^ ILC2 and CD117^−^ ILC2 [17] (Additional file 1: Fig. S1A). The gating of intracellular cytokines was based on the samples without PMA stimulation. (Additional file 2: Fig. S2). For the third assay, the whole blood was incubated with viability kit for 10 min and then surface antibody for another 20 min without stimulation. The surface antibodies included anti-CD45-FITC (clone 2D1, 368508), anti-CD11b-PE/Cy7 (clone CBRM1/5, 301412), anti-CD115-APC (clone 9-4D2-1E4, 347306), anti-CD11c-APC/Cy7 (clone Bu15, 337218), anti-HLA-DR-PerCP/Cy5.5 (clone L243, 307630). After lysis and centrifugation, cell precipitation was resuspended in PBS and for cytometric analysis. In this assay, DCs were defined as CD45^+^HLA-DR^+^CD11c^+^ and macrophages as CD45^+^CD11b^+^CD115^+^ (Additional file 1: Fig. S1B). All antibodies were from Biolengend. Processed samples were analyzed by FCM (FACSAria™ II, BD Biosciences, NJ, USA) and Flow jo™ 10.

### ELISA

Fresh plasma was collected from the blood of 21 healthy subjects, 30 active Mtb-infected patients by centrifuging at 3000r, 10 min. The level of IL-23 in plasma was evaluated by ELISA using human IL-23 ELISA kit (Proteintech, IL, USA) according to manufacturer’s instructions. In brief, 100 μL plasma or standard human IL-23 solution were added to wells and incubated for at 37 °C for 2 h followed by washing with wash buffer for 4 times. Then 100 μL detection antibody was added and incubated at 37 °C for 1 h. After 4 times washing, every well was incubated with 100 μL diluted streptavidin-HRP solution at 37 °C for 40 min and washed by wash buffer for 4 times. Then 100 μL tetramethylbenzidine (TMB) substrate was added and incubated at 37 °C for 20 min in dark, which was stopped by 100 μL stop solution. Then Synergy H1 microplate reader (BioTek, VT, USA) was used to read the plate at 450 nm and 630 nm immediately. The concentration of plasma IL-23 was calculated based on the established standard curve of IL-23.

### Statistical analysis

Statistical analysis and graphs were performed by SPSS 25.0 software (IBM Corporation, NY, USA) and Prism version 7.00 (GraphPad Software, CA, USA). Data were presented as median with interquartile range. Mann–Whitney *U* test was used for comparisons between two groups. Rank correlation was used to test the correlation between plasma IL-23 and the cell proportion. Wilcoxon matched-pairs signed rank test was used to compare the IFN-γ^+^ cells and IL-17^+^ cells out of ILC1 in TB group. Clinical information including age, BMI, symptom score and hemogram indices of TB group was presented as mean ± SD (standard deviation) in Table1, and was compared by Student’s *t* test. Sex and first symptom were compared by Chi-square test. Two-sided *P* < 0.05 was considered significant.

**Table 1:** Clinical comparison of two groups in TB patients

	Total (N = 40)	Low IL-17^+^ ILCs (N = 20)	High IL-17^+^ ILCs (N = 20)	*P* value
Age	49.13 ± 19.49	46.75 ± 17.77	51.50 ± 21.26	0.4481
*Gender*				
Female/male	10/30	3/17	7/13	0.144
BMI	21.50 ± 14.21	21.54 ± 3.45	21.47 ± 4.92	0.9642
First symptom*	26/14	12/7	14/6	0.651
Symptom score	1.58 ± 1.36	1.25 ± 1.29	1.90 ± 1.37	0.1314
*Hemogram indices*				
White blood cells	6.32 ± 1.99	5.78 ± 1.45	6.84 ± 2.31	0.1000
Neutrophils	4.24 ± 1.78	3.75 ± 1.42	4.70 ± 1.99	0.0952
Eosinophils	0.13 ± 0.11	0.17 ± 0.13	0.08 ± 0.05	0.0047
Basophils	0.028 ± 0.02	0.03 ± 0.02	0.03 ± 0.02	0.7881
Lymphocytes	1.38 ± 0.52	1.38 ± 0.46	1.36 ± 0.59	0.9065
Macrophages	0.48 ± 0.24	0.42 ± 0.19	0.54 ± 0.27	0.1386
Red Blood cells	4.36 ± 0.67	4.62 ± 0.63	4.11 ± 0.62	0.0158
Hemoglobin	131.69 ± 21.66	139.42 ± 20.95	124.35 ± 20.14	0.0278
Platelets	223.90 ± 86.65	194.53 ± 45.47	251.80 ± 106.65	0.0373

*First symptom 26/14 means 26 patients started with symptoms while 14 patients with abnormal physical examination

## Results

### Circulatory ILCs were upregulated in active TB disease

**Fig. 1:**
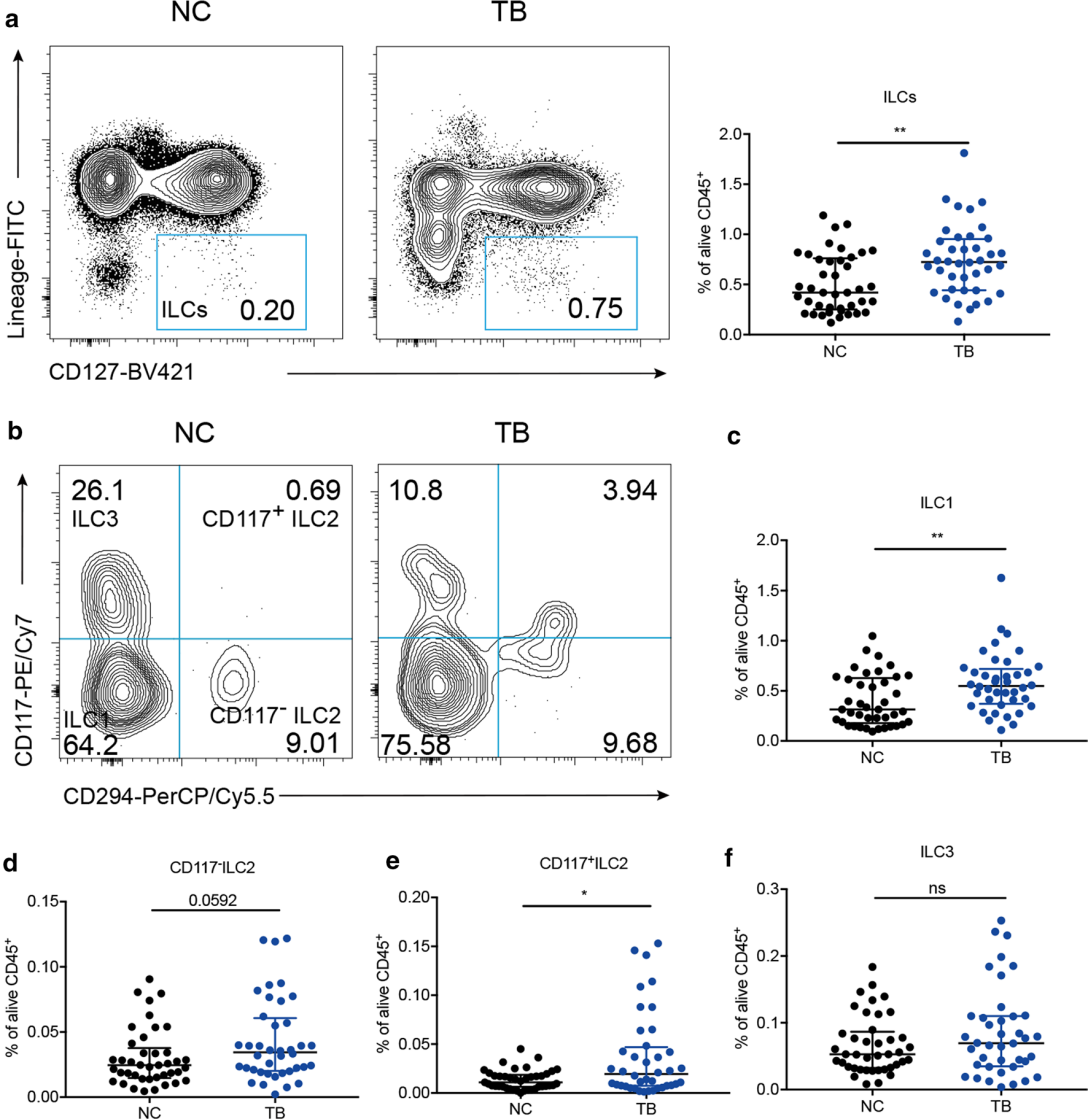
ILCs were upregulated in TB group. Comparison of ILCs (**a**),** b** the typical contour map of two groups, we gave our explanation as followed. ILC1 (**c**), CD117^−^ILC2 (**d**), CD117^+^ILC2 (**e**), ILC3 (**f**) (as proportion of total alive CD45^+^ lymphocytes population) between NC and TB group. *P < 0.05, **P < 0.01

ILCs are an essential cell group reacting quickly to microorganism. We first tested the proportion of ILCs in blood. As shown in Fig. 1a, the median of ILCs in TB group was 0.73%, which was more than that in NC group (0.42%) significantly (*P* = 0.0019). As shown in the typical contour map of two groups (Fig. 1b), we found that ILC1 was increased in active Mtb-infected individuals (0.55%) compared with that in healthy subjects (0.31%, Fig. 1c, *P* = 0.0024). Similarly, CD117^+^ ILC2 in TB group was also increased (0.02% vs. 0.01%, Fig. 1e *P* = 0.0267). CD117^−^ ILC2 and ILC3 were unchanged (Fig. 1d, f). Therefore, circulatory ILCs were upregulated in active TB disease, and the major expansion was in ILC1 and CD117^+^ ILC2 subsets.

### ILCs had increased production of IL-17 in active TB disease

**Fig. 2:**
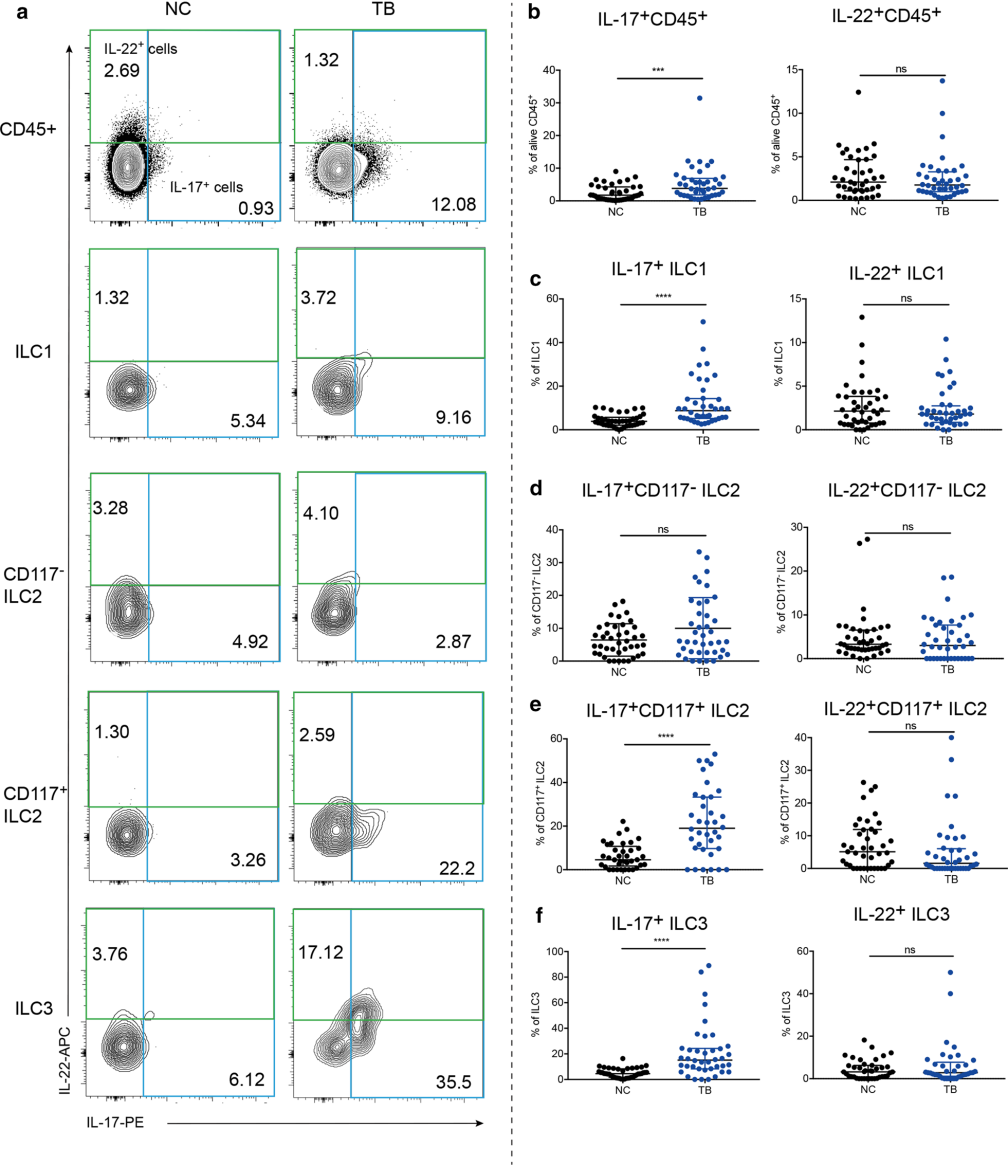
ILCs had increased production of IL-17 in TB group. Contour plot of IL-17/IL-22^+^ cells in different cells (**a**). Comparison in the proportion of IL-17/IL-22^+^ cells out of CD45^+^ lymphocytes (**b**), ILC1 (**c**), CD117^−^ILC2 (**d**), CD117^+^ILC2 (**e**), ILC3 (**f**) between NC and TB group. **P < 0.01, ****P < 0.0001

IL-17 had been reported to be critical for Mtb control [10, 18]. In addition to derivation from Th17 cells, IL-17 from ILCs is also respectable. IL-22 is another important proinflammatory cytokine that mediates host defense against pathogens. In ILCs, ILC3 was the major producer of IL-17 and IL-22, which were demonstrated to be the critical inducers for protective innate immunity against Mtb in lung [3, 12]. Besides, CD117^+^ ILC2 was found to have ILC3-like characteristics—bearing Th17 cytokines-producing potential [8, 19]. Consequently, we analyzed the intracellular expression of IL-17 and IL-22 in the peripheral blood by FCM. In line with our expectation, the median of IL-17^+^ CD45^+^ lymphocytes in TB group was 3.83%, significantly more than that in NC group of 1.76% (Fig. 2b *P* = 0.0006), though the level of IL-22^+^CD45^+^ lymphocytes showed no difference (Fig. 2b). Next, we analyzed the proportion of producing IL-17 and IL-22 cells in ILC subsets. It can be seen that ILC1, CD117^+^ ILC2, ILC3 in TB group all had increased production of IL-17, presenting as the relative frequencies of IL-17^+^ cells (8.79%, 19.01%, 15.15%, respectively) were several times as much as those in NC group (3.87%, 4.57%, 4.55%, Fig. 2c, e, f, *P* < 0.0001). CD117^−^ ILC2 had no difference in producing IL-17 between two groups (Fig. 2d). And all ILC subsets had unchanged expression of IL-22 (Fig. 2c–f). These results together implied an enhanced ability to express IL-17 of circulatory ILCs in active TB disease.

### IL-17 predominated in ILC1 secretion phenotype

**Fig. 3:**
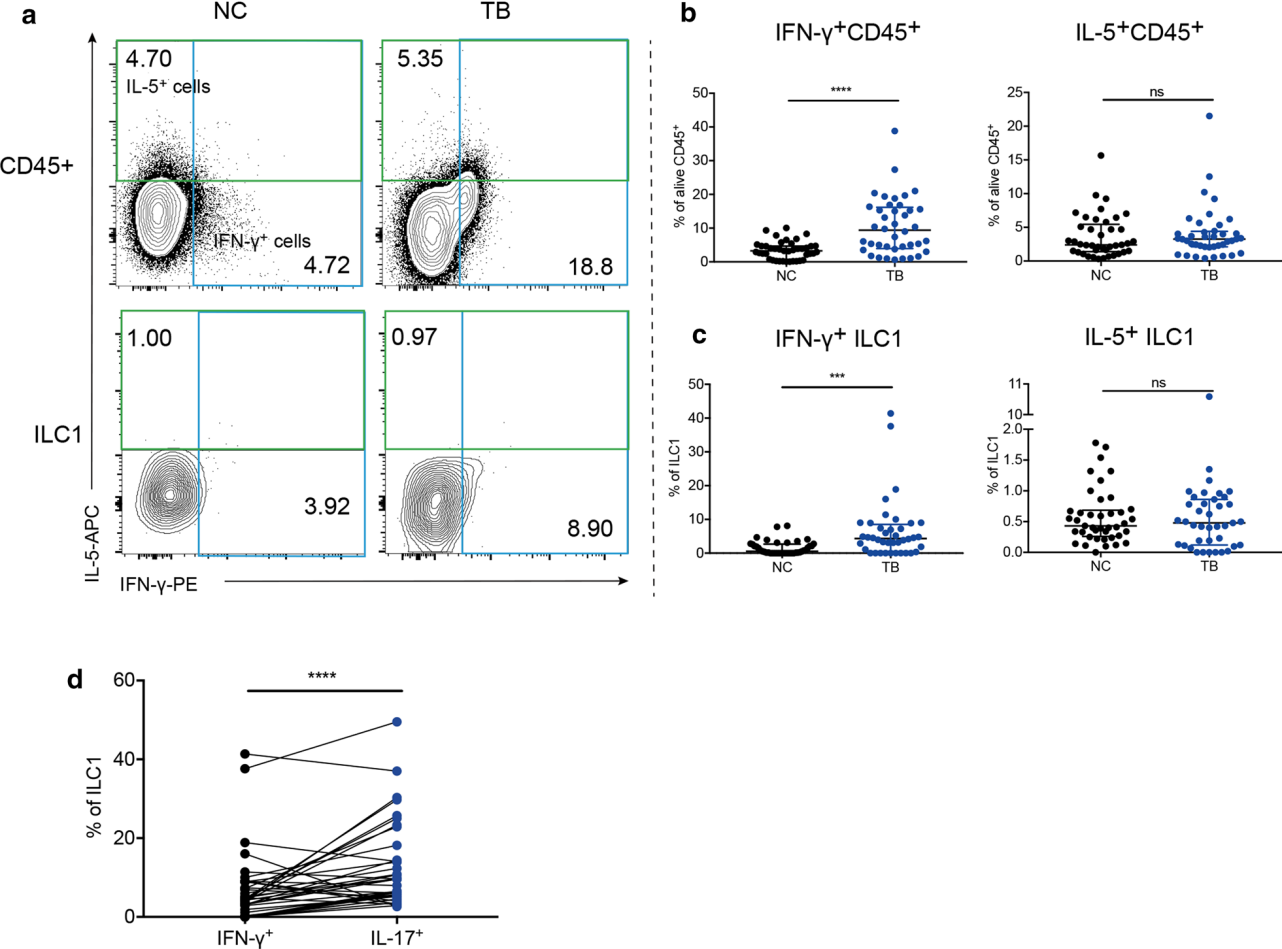
ILC1 had increased expression of IFN-γ in TB group. Contour plot of IFN-γ/IL-5^+^ cells in different cells (**a**). Comparison in the proportion of IFN-γ/IL-5^+^ cells out of CD45^+^ lymphocytes (**b**) and ILC1 (**c**). Comparison in the percentage of IFN-γ^+^ ILC1 and IL-17^+^ ILC1 in TB group by Wilcoxon matched-pairs signed rank test (**d**). *P < 0.05, ***P < 0.001, ****P < 0.0001

Based on the concept that ILC1 is akin to Th1 and ILC2 to Th2, we also tested the function of ILCs to generate Th1 cytokine IFN-γ and Th2 cytokine IL-5. In the whole, the relative frequency of IFN-γ^+^ lymphocytes were 9.41% in TB group, twice more than that in NC group of 3.25% (Fig. 3b, *P* < 0.0001). In ILCs compartment, the production of IFN-γ in ILC1 was dramatically induced in Mtb infection, from 0.56 to 4.37% (Fig. 3c, *P* = 0.0002). CD117^−^ ILC2 had undetectable IFN-γ production (data was not shown); CD117^+^ ILC2 and ILC3 had unchanged expression of IFN-γ (Additional file 3: Fig. S3A–C). As for IL-5, there was no significant difference between two groups in total IL-5^+^ lymphocytes (Fig. 3b). Additionally, ILC1, CD117^−^ ILC2, CD117^+^ ILC2 all had similar expression of IL-5 between two groups (Fig. 3c, Additional file 3: Fig. S3A, D, E). ILC3 had almost undetected expression of IL-5 (data was not shown). Due to that ILC1 had both enhanced expression of IL-17 and IFN-γ, we analyzed which cytokine dominated in ILC1. As shown in Fig. 3d, IL-17^+^ ILC1 (8.79%) was more than IFN-γ^+^ ILC1 (4.37%) in TB group (*P* < 0.0001), which implied a bias of ILC1 into IL-17-expressing phenotype in active TB disease.

### The ability of ILCs to produce IL-17 was correlated to plasma IL-23

**Fig. 4:**
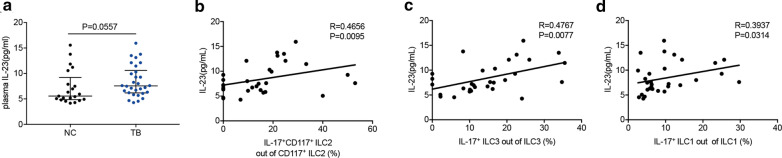
IL-23 was upregulated in TB group. (**a**) Comparison of plasma IL-23 between NC and TB group. The correlation between plasma IL-23 and the percentage of IL-17-producing cells out of CD117^+^ ILC2 (**b**), ILC3 (**c**), ILC1 (**d**)

Previous studies had reported that the production of IL-17 and IL-22 was dependent on stimulation with IL-23 [9, 20]. Consequently, we tested the level of IL-23 in plasma in two groups (21 healthy subjects, 30 active TB patients). The median of plasma IL-23 in two groups was 5.564 pg/mL, 7.551 pg/mL, respectively. Though no statistical meaning was detected, TB group had an increasing tendency of plasma IL-23 than NC group (Fig. 4a, *P* = 0.0557). Then we analyzed the relationship between plasma IL-23 and features of ILCs in TB group by using rank correlation. Firstly, we analyzed whether IL-23 was associated with number of CD117^+^ ILCs in active TB disease. We found the number of CD117^+^ ILC2 and ILC3 had no correlation to plasma IL-23 (Additional file 4: Fig. S4A, B). However, the ability to produce IL-17 of CD117^+^ ILC2 (Fig. 4b, r = 0.4656, *P* = 0.0095), ILC3 (Fig. 4c, r = 0.4767, *P* = 0.0077) were related to plasma IL-23. Surprisingly, the proportion of IL-17^+^ ILC1 to total ILC1 was also correlated to plasma IL-23 (Fig. 4d, r = 0.3937, *P* = 0.0314). Consequently, it could be surmised that the IL-23 may be the potential inducer for production of IL-17 from ILCs in active TB disease.

### DCs were increased in active TB disease

**Fig. 5:**
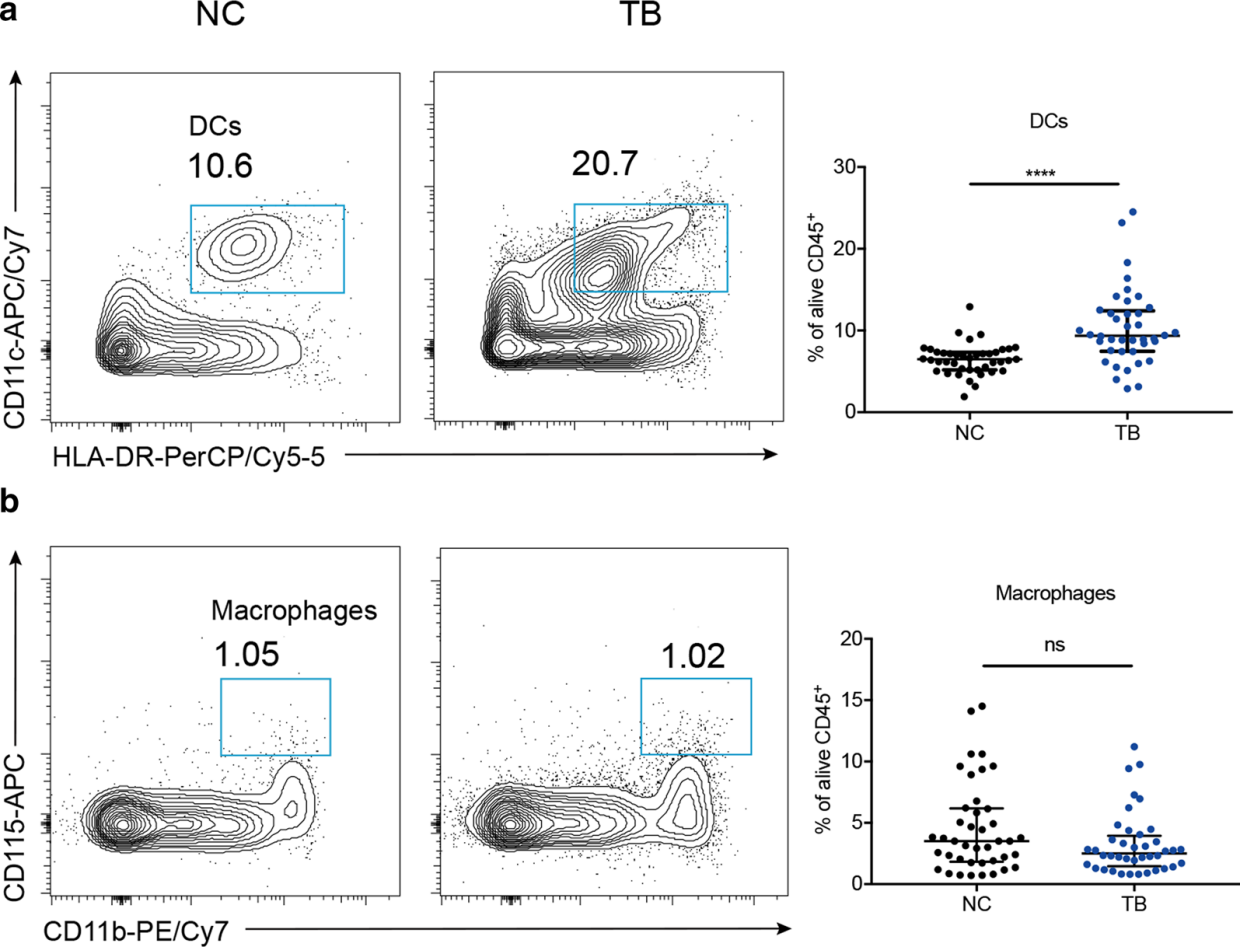
DCs were increased in TB group. Comparison in the percentage of DCs (**a**) and macrophages (**b**) out of CD45^+^ leukocytes. ****P < 0.0001

Antigen-presenting cells (APCs), including macrophages and DCs, were the major sources of IL-23 when they were activated by pathogens. It has been well-established that tissue migratory macrophages and dendritic cells in response to stimulation are derived from monocytes in circulation [21]. During inflammation, circulating monocytes are predestined to become activated macrophages and DCs with some upregulated surface markers [21]. Therefore, we tested whether circulatory monocytes were stirred in active Mtb infection. Based on previous studies [21–23], here we defined HLA-DR^+^CD11c^+^ cells as DCs, CD115^+^CD11b^+^ cells as macrophages (Additional file 1: Fig. S1B) in the third assay. Compared with healthy subjects, patients with active Mtb infection showed higher level of DCs (9.35% vs. 6.49%, Fig. 5a, *P* < 0.0001) but similar level of macrophages (Fig. 5b). It suggested that DCs in vivo was triggered by Mtb for the initial defense.

### IL-17^+^ ILCs was associated with clinical symptom and inflammation

**Fig. 6:**
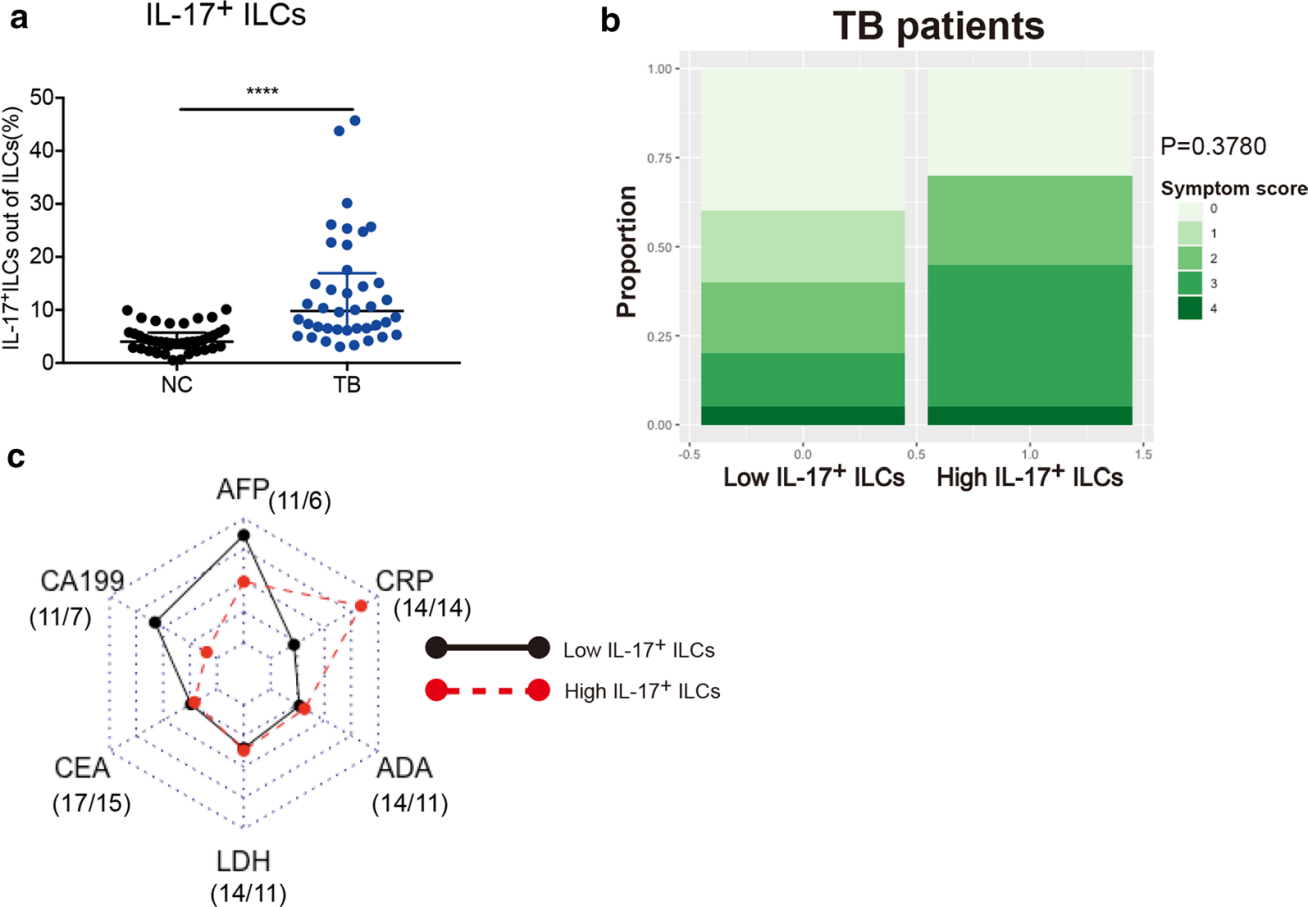
IL-17^+^ ILCs was associated with clinical symptom and inflammation in active TB patients. Comparison in the percentage of IL-17^+^ ILCs out of total ILCs (**a**). Proportion of different symptom score in two groups of TB patients was compared by Chi-square test. **b** Comparison of TB patients with high or low IL-17^+^ ILCs in plasma indices. 11/6 means 11 patients in low IL-17^+^ ILCs group and 6 patients in high IL-17^+^ ILCs group were examined plasma AFP, and so on (**c**). *AFP* alpha-fetoprotein; *CEA* carcino embryonic antigen, *LDH* lactate dehydrogenase; *CRP* C-reactive protein, *ADA* adenosine deaminase. ****P < 0.0001

As mentioned before, ILCs in TB patients exhibited IL-17-expressing phenotypes compared with NC group, we again compared the proportion of IL-17^+^ ILCs out of total ILCs between two groups. As shown in Fig. 6a, ILCs in TB group had evident upregulated expression of IL-17 (9.81% vs. 4.00%, *P* < 0.0001). We then analyzed whether this phenotype was associated with clinical symptoms and laboratory measurements. According to the median of IL-17^+^ ILCs, we divided TB patients into two groups, and the clinical information was listed in Table1. Clinical symptoms including cough, sputum, fever, purulent sputum, bloodshot sputum, chest pain and loss of weight were scored 1 unanimously and then added up to a total symptom score. Patients with higher frequency of IL-17^+^ ILCs tend to have more white blood cells (*P* = 0.1000) and neutrophils (*P* = 0.0952) but less eosinophils (*P* = 0.0047). Moreover, this group had obvious poor clinical condition with lower red blood cells (*P* = 0.0158), hemoglobin level (*P* = 0.0278) but higher platelet level (*P* = 0.0373). Though the proportion of patients reporting clinical symptoms had no significant difference between two groups (Fig. 6b, *P* = 0.3780), patients with higher IL-17^+^ ILCs still tended to get higher symptom scores (Table1, *P* = 0.1314). Because of the similar lung mass or nodules in pulmonary images between lung TB and lung cancer, some patients were examined plasma cancerous indices such alpha-fetoprotein (AFP), CA199, carcino embryonic antigen (CEA), and inflammatory indices such as lactate dehydrogenase (LDH), C-reactive protein (CRP), adenosine deaminase (ADA) for differential diagnosis. We found that patients with higher IL-17^+^ ILCs tended to harbor higher CRP but lower CA199 and AFP. The specific marker for Mtb infection ADA was not different between two groups (Fig. 6c). These results indicated that IL-17^+^ ILCs was associated with inflammatory status and poor condition of active TB patients.

## Discussion

Mtb infection now is still the leading cause of death and a huge burden economically from an infectious disease worldwide. Despite the invention of BCG to reduce Mtb contagion in childhood, TB population cannot be curbed due to variable efficacy of BCG in protecting adults from infection. Much efforts had been made to explore Mtb-mediated immune invasion in human, with the hope of establishing a targeted immune therapy for TB treatment [16]. Under this circumstances, innate immunity gains growing focus for its powerful ability of recognition, presentation and elimination of Mtb, as well as its indispensable role for activation of adaptive immunity. ILCs, as an important innate cell responding to pathogens before adaptive immunity develops, was considered to protect from various infectious diseases. And its role in Mtb infection was also appealing much discussion and exploration.

Recently, evidence had been discovered that ILCs were important for immunity against Mtb. ILC2 had been reported to accumulate in BCG-immunized lung with unclear function [4]. And a declined circulating ILCs was found in Mtb patients (n = 22) due to boosted migration into infected sites comparing with healthy controls (n = 19) [3]. Contrarily, in our study, Mtb patients presented an upregulated total ILCs and evidently increased CD117^+^ ILC2 and ILC1 in peripheral blood, suggesting an expansion of circulatory ILCs in Mtb-infected subjects. This difference may result from the sample size as well as the different marker for cell gating.

It is well-established that expression of IFN-γ from T cells is the major determinant of TB immunity [16]. It is also supported by our results that active Mtb-infected patients had increased IFN-γ^+^ lymphocytes. A research reported an upregulated secretion of IFN-γ from ILC1 with BCG vaccination [4], consistent with upregulated IFN-γ^+^ ILC1 in our TB group. However, our results underlined that in Mtb infection, IL-17-expressing phenotype predominated in ILCs.

ILCs are largely tissue-resident cells which enriched in mucosa such as skin and lung, supporting barrier immunity [24, 25]. Though it was widely acknowledged that increase of ILCs in organ during systemic inflammation resulted from local expansion, a modest recruitment of circulatory ILC2 into inflammatory tissues in chronic inflammation phase was still observed [26]. In addition, circulatory ILCs changes were also reported in psoriasis [27] and inflammatory bowel disease patients [28]. Consequently, we surmised that circulating ILCs could reflect host inflammatory status during pulmonary tuberculosis, contributing to understanding of ILCs in Mtb infection. ILCs can change phenotype and function promptly in response to stimulating signals provided by peripheral tissues [29], by cell proliferation, mobilization and conversion among three ILC subsets [8, 9, 30]. Studies indicated CD117 could mark distinctly different subpopulation of ILC2 in circulation [19, 31]. Expression of CD117 was coincided with the expression of retinoid-related orphan nuclear receptor γt (RORγt), which was a typical transcriptional factors of ILC3 [8]. In vitro, CD117^+^ ILC2 had ILC3-like phenotype with apparent IL-17 production in presence of ILC3-prompting conditions while CD117^−^ ILC2 maintain the traditional ILC2 identity [8, 19]. ILC1 could also retain ILC3-like characteristics in stimulation with IL-23. In a study of non-small lung cancer, tumor cell-derived IL-23 was able to convert ILC1 into ILC3 [9]. Our study showed a manifest production of IL-17 from ILC1, CD117^+^ ILCs including ILC3 and CD117^+^ ILC2 in Mtb infection, suggesting a pro-inflammatory function of ILCs in circulation. But whether this augmentation resulted from conversion among ILC subsets or from mobilization or proliferation is unknown.

**Fig. 7:**
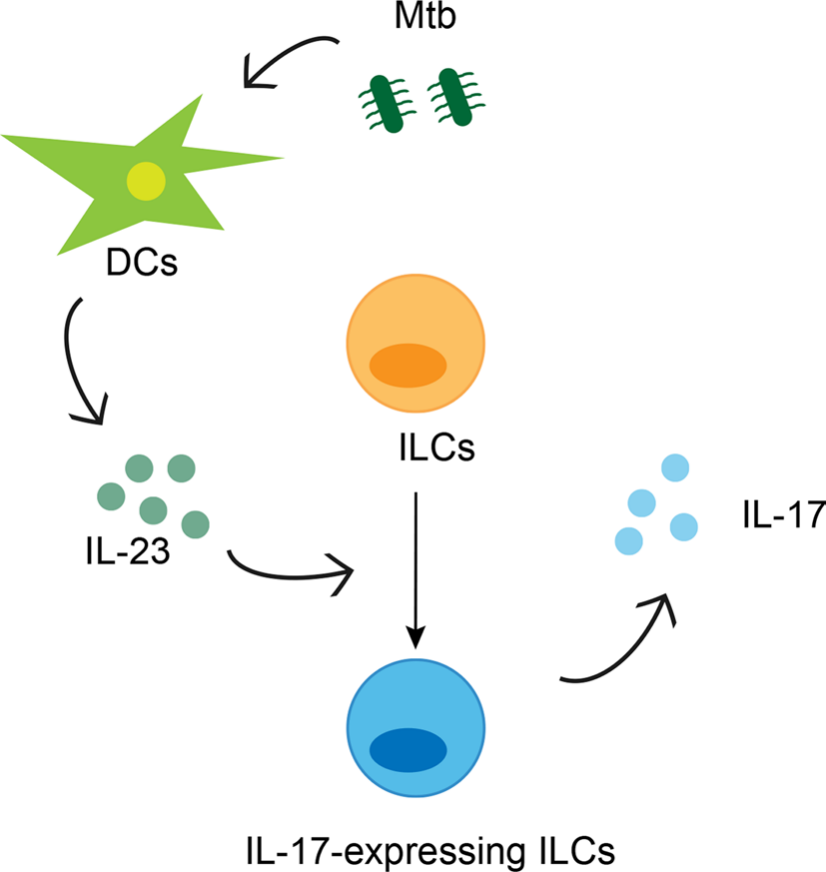
Probable mechanism of ILCs functional changes in active Mtb infection. In Mtb infection, DCs were activated to produce IL-23. The increase of IL-23 promoted the differentiation of ILCs into IL-17-expressing phenotype, thereby increasing the production of IL-17 and participating in the inflammation

IL-23 was the well-established cytokines that is essential for production of IL-17 and IL-22 [9, 20], especially DCs/macrophages-derived IL-23 was acknowledged to accelerate when stimulated by pathogens [20], in accordance with soaring DCs in active Mtb-infected patients in our study. And the dependency of IL-23 to the majority of IL-17 response in Mtb infection had been discovered [13]. Studies had demonstrated that IL-23 alone was sufficient to activate the production of IL-17 from ILC3, and addition of IL-1β and IL-7 was able to stimulate the proliferation of ILC3 [32, 33]. Consistently, our study found a correlation between plasma IL-23 and the ability to produce IL-17 of ILC3, CD117^+^ ILC2, but not the number of these cells. Of interest, plasma IL-23 was also related to the capacity to secret IL-17 of ILC1. We speculated that in active Mtb infection, DCs were activated to secrete IL-23, which promote ILCs differentiating into IL-17-expressing phenotype (Fig. 7). But this conjecture should be verified further. Whether IL-17 is a protective factor is controversial. Study reported that IL-17 could enhance the activity of matrix metalloproteinase-3, which mediated pulmonary tissue destruction in TB [15], while others found that after vaccination, IL-17^+^CD4^+^ T cells populated in lung contributed to expression of IFN-γ from CD4^+^ T cells, restricting Mtb proliferation [11]. In our study, we found IL-17^+^ ILCs was related to systemic inflammation and clinical condition. Its detailed role in TB immunity and clinical indication will be explored later.

## Supplementary Information

**Additional file 1: Figure S1.** Gating strategies in three assays. ILCs and subsets were measured by flow cytometry. Production of IL-17, IL-22, IL-5 and IFN-γ was measured in alive CD45+ cells and innate lymphoid cells subsets (A). Dendritic cells and macrophages were tested by flow cytometry (B). FSC-A, forward scatter-area; SSC-A, side scatter-area; FSC-H, forward scatter-height, BV, Brilliant Violet; FITC, fluorescein isothiocyanate; PerCP-Cy5-5, peridinin–chlorophyll–protein–cyanin5.5; PE-Cy7, phycoerythrin cyanine 7; APC-Cy7, allophycocyanin cyanin7; PE, phycoerythrin; APC, allophycocyanin; IFN-γ, interferon-γ; ILCs: innate lymphoid cells.

**Additional file 2: Figure S2.** Gating of intracellular cytokines.

**Additional file 3: Figure S3.** The production of IFN-γ and IL-5 in ILCs. The production of IFN-γ and IL-5 in CD117- ILC2, CD117+ ILC2 and ILC3 (A). Comparison in the proportion of IFN-γ-producing cells out of CD117+ ILC2 (B) and ILC3 (C). Comparison in the proportion of IL-5-producing cells out of CD117- ILC2 (D), CD117+ ILC2 (E). NC: normal control group; TB: Mtb-infected group. ILC: innate lymphoid cell.

**Additional file 4: Figure S4.** The correlation between plasma IL-23 and CD117+ ILC2, ILC3 in TB group. The correlation between plasma IL-23 and CD117+ ILC2 (A) (as the percentage of total CD45+ lymphocytes). The correlation between plasma IL-23 and ILC3 (B) (as the percentage of total CD45+ lymphocytes).

## Data Availability

Materials and data are available upon request.

## Abbreviations

ADA: Adenosine deaminase
AFP: Alpha-fetoprotein
APCs: Antigen-presenting cells
BCG: Bacille Calmette–Guérin
CEA: Carcino embryonic antigen
CRP: C-reactive protein
CXCL13: C-X-C motif ligand 13
CXCR5: C-X-C chemokine receptor type 5
DCs: Dendritic cells
ELISA: Enzyme-linked immunosorbent assay
FCM: Flow cytometry
IFN-γ: Interferon-γ
ILCs: Innate lymphoid cells
LDH: Lactate dehydrogenase
Mtb: *Mycobacterium tuberculosis*
NK cells: Natural killer cells
RORγt: Retinoid-related orphan nuclear receptor γt
TB: Tuberculosis
Th1: Type 1 immune response
