# Supplementary material for: Innate lymphoid cells exhibited IL-17-expressing phenotype in active tuberculosis disease
Source: BMC Pulm Med. 2021 Oct 12;21:318. doi: 10.1186/s12890-021-01678-1 (PMC8513179; doi:10.1186/s12890-021-01678-1)
Supplement: Supplementary file 1 — Additional file 1: Figure S1. Gating strategies in three assays. ILCs and subsets were measured by flow cytometry. Production of IL-17, IL-22, IL-5 and IFN-γ was measured in alive CD45+ cells and innate lymphoid cells subsets (A). Dendritic cells and macrophages were tested by flow cytometry (B). FSC-A, forward scatter-area; SSC-A, side scatter-area; FSC-H, forward scatter-height, BV, Brilliant Violet; FITC, fluorescein isothiocyanate; PerCP-Cy5-5, peridinin–chlorophyll–protein–cyanin5.5; PE-Cy7, phycoerythrin cyanine 7; APC-Cy7, allophycocyanin cyanin7; PE, phycoerythrin; APC, allophycocyanin; IFN-γ, interferon-γ; ILCs: innate lymphoid cells. [file 12890_2021_1678_MOESM1_ESM.docx]

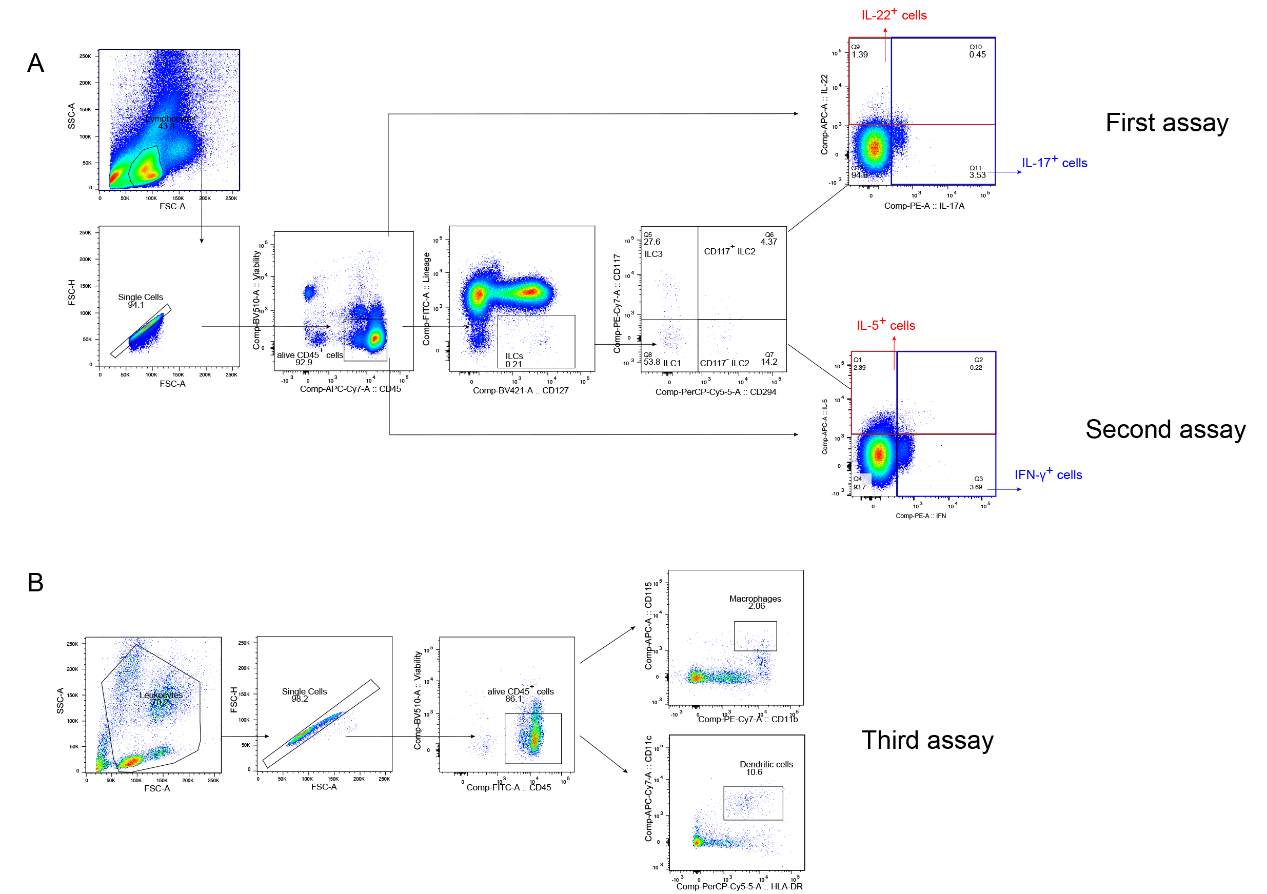


**Additional file 1: Figure S1.** Gating strategies in three assays.

ILCs and subsets were measured by flow cytometry. Production of IL-17, IL-22, IL-5 and IFN-γ was measured in alive CD45^+^ cells and innate lymphoid cells subsets (A). Dendritic cells and macrophages were tested by flow cytometry (B). FSC-A, forward scatter-area; SSC-A, side scatter-area; FSC-H, forward scatter-height, BV, Brilliant Violet; FITC, fluorescein isothiocyanate; PerCP-Cy5-5, peridinin–chlorophyll–protein–cyanin5.5; PE-Cy7, phycoerythrin cyanine 7; APC-Cy7, allophycocyanin cyanin7; PE, phycoerythrin; APC, allophycocyanin; IFN-γ, interferon-γ; ILCs: innate lymphoid cells.
