# Supplementary figures and images for: Innate lymphoid cells exhibited IL-17-expressing phenotype in active tuberculosis disease
Source: BMC Pulm Med. 2021 Oct 12;21:318. doi: 10.1186/s12890-021-01678-1 (PMC8513179; doi:10.1186/s12890-021-01678-1)

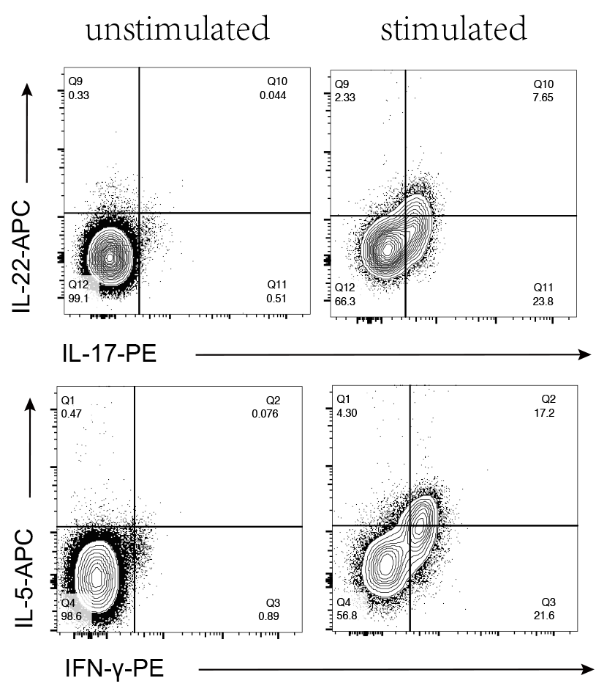


**Additional file 2: Figure S2.** Gating of intracellular cytokines

Supplement: Supplementary file 2 — Additional file 2: Figure S2. Gating of intracellular cytokines. [file 12890_2021_1678_MOESM2_ESM.docx]
