# Supplementary material for: Innate lymphoid cells exhibited IL-17-expressing phenotype in active tuberculosis disease
Source: BMC Pulm Med. 2021 Oct 12;21:318. doi: 10.1186/s12890-021-01678-1 (PMC8513179; doi:10.1186/s12890-021-01678-1)
Supplement: Supplementary file 3 — Additional file 3: Figure S3. The production of IFN-γ and IL-5 in ILCs. The production of IFN-γ and IL-5 in CD117- ILC2, CD117+ ILC2 and ILC3 (A). Comparison in the proportion of IFN-γ-producing cells out of CD117+ ILC2 (B) and ILC3 (C). Comparison in the proportion of IL-5-producing cells out of CD117- ILC2 (D), CD117+ ILC2 (E). NC: normal control group; TB: Mtb-infected group. ILC: innate lymphoid cell. [file 12890_2021_1678_MOESM3_ESM.docx]

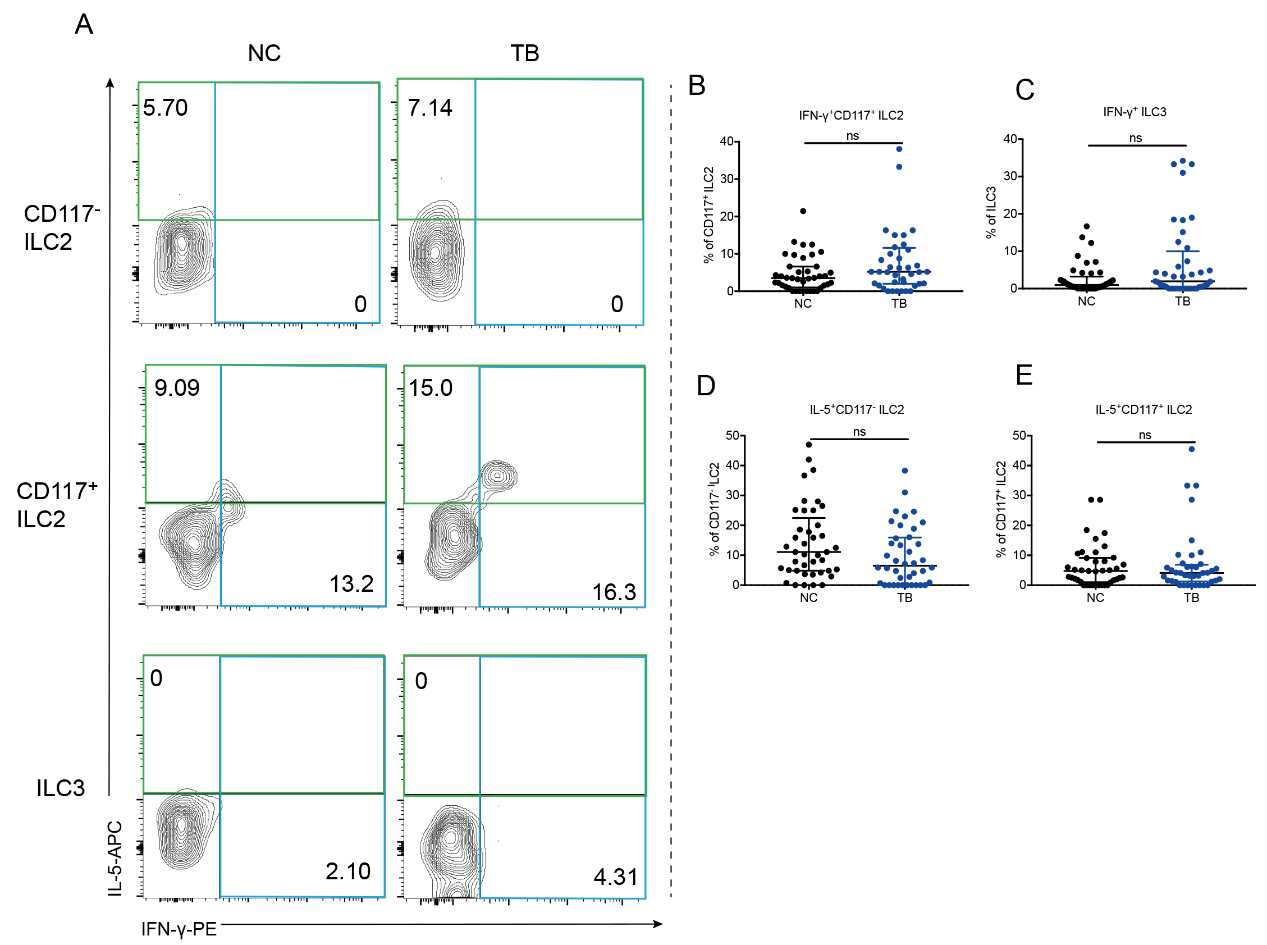


**Additional file 3: Figure S3.** The production of IFN-γ and IL-5 in ILCs

The production of IFN-γ and IL-5 in CD117^-^ ILC2, CD117^+^ ILC2 and ILC3 (A). Comparison in the proportion of IFN-γ-producing cells out of CD117^+^ ILC2 (B) and ILC3 (C). Comparison in the proportion of IL-5-producing cells out of CD117^-^ ILC2 (D), CD117^+^ ILC2 (E). NC: normal control group; TB: Mtb-infected group. ILC: innate lymphoid cell.
