# Supplementary material for: Innate lymphoid cells exhibited IL-17-expressing phenotype in active tuberculosis disease
Source: BMC Pulm Med. 2021 Oct 12;21:318. doi: 10.1186/s12890-021-01678-1 (PMC8513179; doi:10.1186/s12890-021-01678-1)
Supplement: Supplementary file 4 — Additional file 4: Figure S4. The correlation between plasma IL-23 and CD117+ ILC2, ILC3 in TB group. The correlation between plasma IL-23 and CD117+ ILC2 (A) (as the percentage of total CD45+ lymphocytes). The correlation between plasma IL-23 and ILC3 (B) (as the percentage of total CD45+ lymphocytes). [file 12890_2021_1678_MOESM4_ESM.docx]

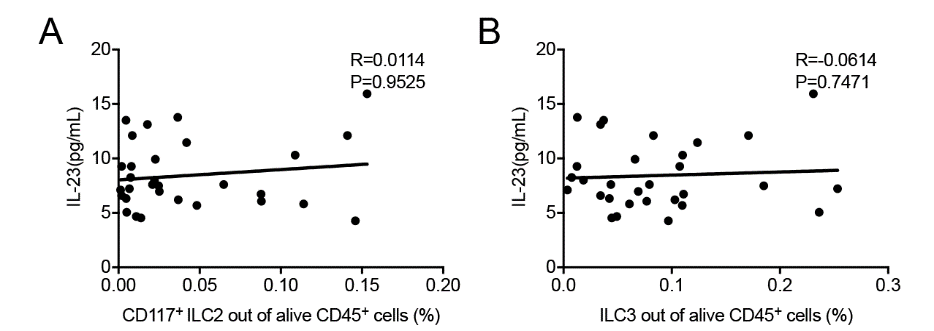


**Additional file 4: Figure S4.** The correlation between plasma IL-23 and CD117^+^ ILC2, ILC3 in TB group

The correlation between plasma IL-23 and CD117^+^ ILC2 (A) (as the percentage of total CD45^+^ lymphocytes). The correlation between plasma IL-23 and ILC3 (B) (as the percentage of total CD45^+^ lymphocytes).
